# A Triplex Ultrasound Evaluation of Preclinical Changes in Type 2 Diabetes in Foot Arteries

**DOI:** 10.7759/cureus.23119

**Published:** 2022-03-13

**Authors:** Rawan I Ali, Awadia G Suliman, Ahmed Abdelrahim, Moawia Gameraddin

**Affiliations:** 1 Department of Diagnostic Radiologic Technology, Faculty of Radiology Science and Medical Imaging, Alzaiem Alazhari University, Khartoum, SDN; 2 Department of Diagnostic Radiologic Technology, College of Applied Medical Sciences, Taibah University, Medina, SAU

**Keywords:** resistive index, diameters, posterior tibial artery, dorsalis pedis artery, diabetes mellitus

## Abstract

Background and objective: Type 2 diabetes mellitus (T2DM) is a significant health problem that is becoming more prevalent worldwide. This study aimed to assess hemodynamic and morphological parameters in diabetic patients' foot arteries and compare them to those obtained in asymptomatic control group.

Materials and methods: This is a cross-sectional case-control study. B-mode ultrasound, color Doppler, and pulse wave Doppler were conducted to assess the dorsalis pedis arteries (DPAs) and posterior tibial arteries (PTAs). The morphological, total vascular diameter, wall thickness, and flow Doppler indices were measured. A total of 200 hundred participants were selected randomly using a random sampling technique. One hundred diabetic patients and 100 non-diabetic persons were determined.

Results: In diabetic patients, the overall grayscale diameter and wall thickness of foot arteries were statistically significantly larger than the asymptomatic group in the right DPA (p<0.01), left DPA (p<0.001), right PTA (p<0.001), and left PTA (p<0.001). In the diabetic group, the level of hemoglobin A1c (HbA1c) was positively correlated with blood flow resistive index (RI) in the right DPA (r=0.839; p<0.001), left DPA (r=0.801; p<0.001), right PTA (r=0.801; p<0.001), and left PTA (r=0.801; p<0.001). No significant differences were noted in both groups in blood flow Doppler parameters - pulsatility index (PI) and resistive index (RI).

Conclusion: Overall grayscale diameters of foot arteries are larger in the diabetes group than in the control group, indicating arterial wall thickening as an early indicator of diabetes-related alterations. PI of both DPA and RI of right DPA were increased in diabetic patients more than the control group. The level of glycosylatedhemoglobin A1c (HbA1c) was strongly linked with the blood flow resistive index in diabetes patients.

## Introduction

T2DM affects a large population, and the WHO predicts an increase in the number of adults with diabetes [1]. Type 2 diabetes mellitus (T2DM) is the most common form of diabetes. Since most diabetic patients (around 90-95%) suffer from type 2 diabetes mellitus (T2DM), the numbers will increase to 693 million by 2045 [1-3]. This disease affects multiple organs, particularly the blood vessels.

The most serious complication of type 2 diabetes is atherosclerosis, which results from a multistep process leading to cardiovascular disease, characterized by high morbidity and mortality [4]. Type 2 diabetes mellitus leads to an increase in the stiffness of peripheral vessels due to early-onset atherosclerosis [5]. A major manifestation of arterial stiffening is a change in the cyclic arterial pressure as it leads to increase systolic arterial pressure and decreased diastolic blood pressure [6].

There are various techniques and methods to assess the lower limb arteries, such as pulse measurements, ankle-brachial index (ABI), and the primary measure used in clinical diagnostics, Doppler ultrasound, conventional arteriography, CT angiography, and magnetic resonance angiography (MRA). Triplex ultrasound is considered the most crucial imaging method as it is non-invasive and provides an accurate hemodynamic evaluation of the foot arteries [7].

The purpose of this study was to examine the hemodynamic and morphological characteristics of two-foot arteries; posterior tibial artery (PTA) and dorsalis pedis artery (DPA) in patients with type 2 diabetes mellitus (DM) using triplex ultrasound and to compare the findings to those conducted in a control group.

## Materials and methods

This was a cross-sectional case-control study, conducted in Shifa Alaleel Specialized Hospital, from February 2019 to October 2021. The study included 100 T2DM subjects as a diabetes group and 100 asymptomatic non-diabetic subjects as a control group. The T2DM group patients were well-controlled (HbA1c less than 7%) in stage I of the Fontaine classification for peripheral artery disorders, which means they had no clinical symptoms and normal ABI results. The participants in the control group were asymptomatic, with no limb ischemia or medical condition affecting the peripheral arteries, ABI normal values, normal glucose and lipid metabolism, no family history of diabetes or hypertension, and no smoking history. The exclusion criteria included patients with lower limb arterial disease, smoking habit, hypertension, and any other conditions affecting the arterial Doppler flow measurements. Scientific Research and Ethics Committee of Faculty of Radiological Science and Medical Imaging in Alzaiem Alazhari University granted an unconditional approval on February 26, 2019 (#AAU-FRSMI-0019/13). Written consent was taken from the study participants; then the data was collected by a data collection sheet designed to satisfy all the demographic and sonographic data variables using an ultrasound machine for scanning.

Statistical analysis

The data were analyzed using Statistical Package for Social Sciences (SPSS) statistics version 23 (Armonk, NY: IBM Corp.), the frequency performed for categorical variables, then descriptive statistic for continuous variables. Student's t-test was applied to compare the Doppler parameters and the equality of means in diabetes and control groups. Cross-tabulation was performed using chi-square test, while Pearson's correlation was used to correlate between the quantitative variables. P-values lesser than 0.05 were considered significant.

The Doppler and sonographic procedure

**Figure 1:**
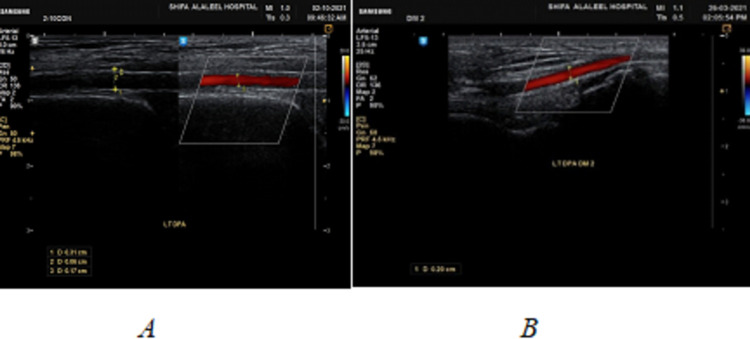
A longitudinal portion of a Doppler color sonography of the left DPA demonstrates blood flow lumen diameter measurement (A) in the control group and (B) in the diabetes group. DPA: dorsalis pedis artery

**Figure 2:**
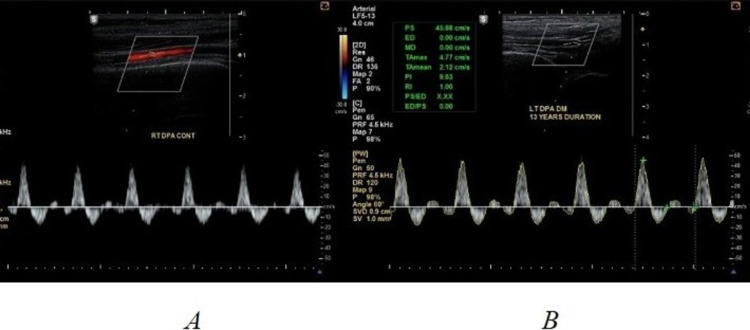
The image is showing (A) right DPA pulse wave of asymptomatic participant in the control group and (B) left DPA pulse wave of a diabetic patient with 13 years’ duration of diabetes. DPA: dorsalis pedis artery

Triplex ultrasound examination has been performed using a Samsung H60 machine (Seoul, South Korea: Samsung) with a linear transducer with a primary frequency of 7.5 MHz. Doppler investigations of the DPA and PTA were conducted in a supine position with the limb flexed at an angle of 90°. The following ultrasound assessments of DPAs and PTAs were conducted: (i) B-mode assessment to assess the external diameter of foot arteries and wall thickness of the vessels; (ii) color Doppler imaging to evaluate the diameters of color vessels flow (Figure 1); (iii) spectral Doppler imaging to evaluate the resistivity and pulsatility of blood flow, such as pulsatility index (PI) and resistance index (RI), in addition to the shape of the waveform (Figure 2).

**Figure 3:**
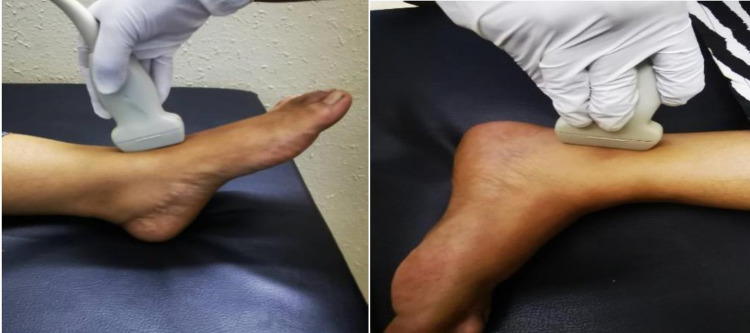
The image shows the sonographic scanning technique for DPA and PTA. The photo was taken from a participant in the study.

The machine was set for Doppler imaging of the lower limb arteries, DPA, and PTA. The patient was investigated in a supine position with the US transducer traced from the anterior ankle to the dorsal foot to evaluate the DPA. After identifying the foot arteries, diameter and wall thickness were measured in B-mode, color Doppler was activated, and flow lumen diameter was measured. For examining the PTA, the participants were investigated in lateral decubitus, and the probe was placed on the posterior aspect of the medial malleolus. The diameter and wall thickness of the arteries were measured in B-mode, then color Doppler activated, and flow lumen diameter was measured. Finally, a pulse wave was set to calculate the PI and RI values (Figure 3).

## Results

**Figure 4:**
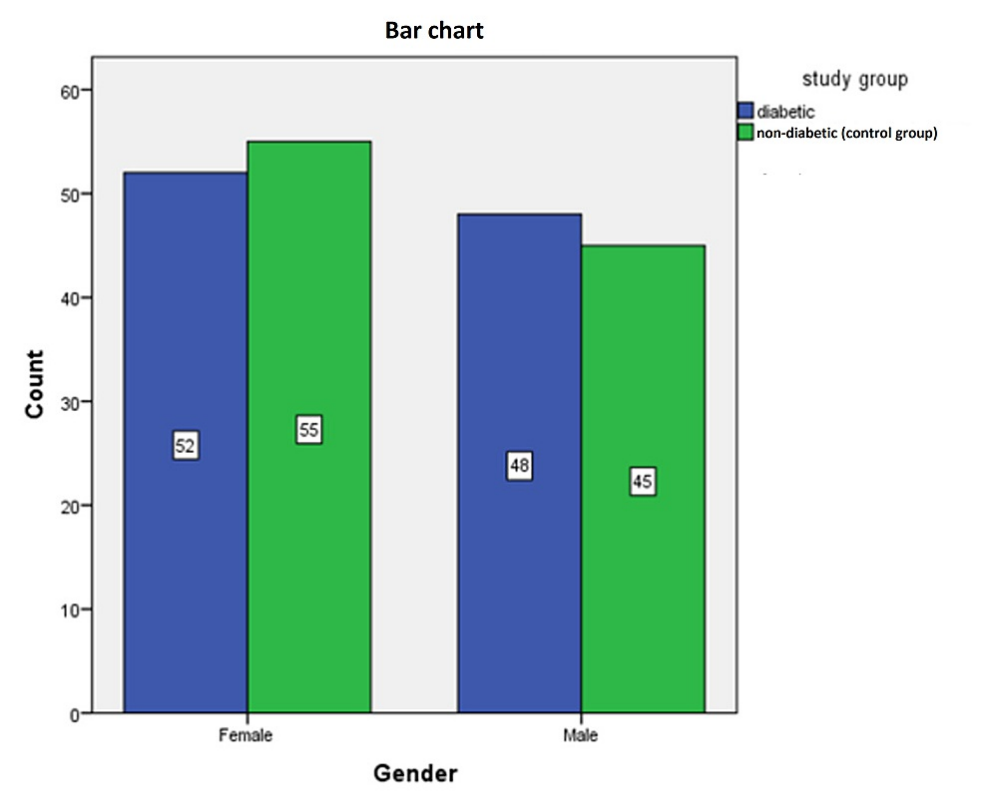
Frequency distribution of gender in the study groups.

This case-control non-interventional study was conducted in Khartoum, Sudan, to assess the foot arteries in preclinical diabetic patients using grayscale and Doppler ultrasound. The study included 200 patients, 100 diabetic persons in a diabetes group and 100 non-diabetic persons in a control group. There were no significant differences concerning demographic characteristics, such as mean age, gender, and ankle-brachial index (ABI) (Figure 4, Table 1). The mean age and ankle-brachial indexes are 56.13 and 55.26 years and 1.08±0.08, 1.08±0.07 for right and left ABI in diabetes and control groups, respectively. Significant differences were found in BMI for diabetes and control groups (30.22±4.11 vs. 25.59±1.86, respectively; p≤0.01). The mean duration and HbA1c of the diabetes group were 10.35±8.50 years and 6.63±0.45 mmol/mol, respectively (Table 1).

**Table 1:** Demographic characteristics of diabetes group (diabetic patients) and control group (non-diabetic patients). ABI: ankle-brachial index; HbA1c: hemoglobin A1c

Demographic characteristics	Diabetes group (mean±SD)	Control group (mean±SD)	p-Value
Age (years)	56.13±16.06	55.26±14.51	0.688
BMI (kg/cm^2^)	30.22±4.11	25.59±1.86	<0.001
HbA1c (mmol/mol)	6.63±0.45	-	-
Duration (years)	10.35±8.50	-	-
ABI right	1.08±0.08	1.08±0.07	1.000
ABI left	1.08±0.08	1.08±0.07	1.000

**Figure 5:**
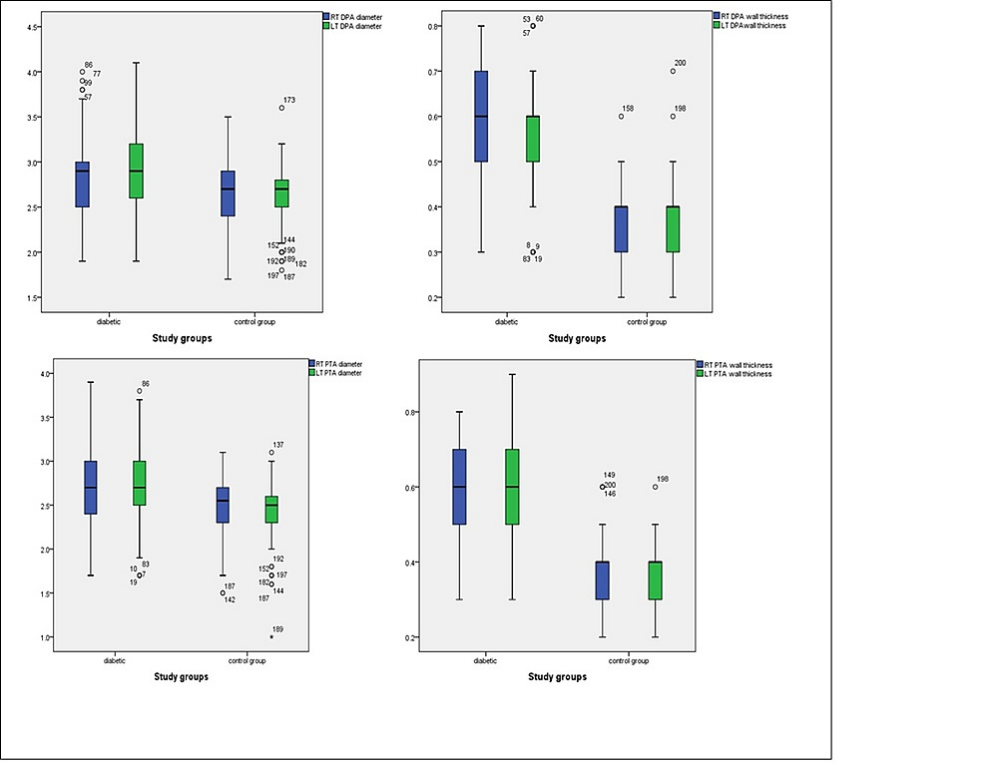
Mean arterial diameters and wall thickness of DPA and PTA of both sides. DPA: dorsalis pedis artery; PTA: posterior tibial artery

The blood vessel diameter and arterial thickness were significantly larger in the diabetes group as compared to the control group (Table 2, Figure 5). There were no significant differences in color Doppler lumen diameters between cases and controls (Table 2).

**Table 2:** Comparison of diameter and Doppler indexes of DPA and PTA in diabetes and control groups. DPA: dorsalis pedis artery; PTA: posterior tibial artery; PI: pulsatility index; RI: resistance index p<0.05 is significant.

Diameters, thickness, and Doppler indexes of DPA and PTA	Diabetes group (mean±SD)	Control group (mean±SD)	p-Value
Right DPA grayscale diameter (mm)	2.81±0.44	2.64±0.36	0.003
Left DPA grayscale diameter (mm)	2.90±0.49	2.63±0.31	<0.001
Right DPA lumen diameter Doppler (mm)	2.03±0.37	2.04±0.29	0.754
Left DPA lumen diameter Doppler (mm)	2.05±0.38	2.03±0.29	0.666
Right DPA wall thickness (mm)	0.58±0.15	0.38±0.08	<0.001
Left DPA wall thickness (mm)	0.56±0.12	0.38±0.08	<0.001
Right DPA PI	8.50±2.92	8.40±2.39	0.810
Left DPA PI	8.65±2.72	8.62±2.48	0.929
Right DPA RI	0.94±0.13	0.93±0.08	0.418
Left DPA RI	0.93±0.13	0.93±0.08	0.574
Right PTA diameter (mm)	2.70±0.46	2.49±0.31	<0.001
Left PTA diameter (mm)	2.67±0.45	2.42±0.34	<0.001
Right PTA lumen diameter Doppler (mm)	1.97±0.33	1.99±0.29	0.588
Left PTA lumen diameter Doppler (mm)	1.93±0.33	1.93±0.25	0.981
Right PTA wall thickness (mm)	0.58±0.15	0.38±0.08	<0.001
Left PTA wall thickness (mm)	0.56±0.13	0.38±0.08	<0.001
Right PTA PI	8.49±2.63	8.61±2.47	0.730
Left PTA PI	8.59±2.63	8.57±2.47	0.951
Right PTA RI	0.93±0.13	0.93±0.08	0.577
Left PTA RI	0.94±0.14	0.93±0.09	0.574

The PI of both DPA and RI of right DPA increased in diabetic patients compared to the controls without significant difference (p>0.05) and so the RI in the left PTA (Table 2). The PI values of the right PTA decreased in diabetes group compared to the control group, while in left PTA the values increased in diabetes group compared to control group without significant difference (right PTA=8.49±2.63, left PTA=8.59±2.63 in diabetes group vs. right PTA=8.61±2.47, left PTA=8.57±2.47 in control group, respectively; p>0.05). The shape of waveforms of both arteries was evaluated. The waveform is triphasic in appearance in 86% of diabetic patients and biphasic in the rest 14% while in the control group, the shape of the waveform is triphasic high resistance in all cases (100%) as shown in Table 3. The study found that there was a strong significant correlation between HbA1c and blood resistive index in both DPA and PTA in diabetic patients (r=0.839, p≤0.01, r=0.801, p<0.01, respectively, for RI of right DPA and HbA1c and RI of left DPA, and both PTAs) (Table 4).

**Table 3:** The shape of a waveform in diabetes and control groups.

Shape of waveform	Diabetes group (frequency)	Control group (frequency)	p-Value
Biphasic	14	0	<0.001
Triphasic	86	100	
Total	100	100	

**Table 4:** Correlation between HbA1c and Doppler blood flow parameters of both foot arteries in diabetic patients. DPA: dorsalis pedis artery; PTA: posterior tibial artery; PI: pulsatility index; RI: resistance index; HbA1c: hemoglobin A1c *The value is significant at <0.01.

		Right DPA PI	Right DPA RI	Left DPA PI	Left DPA RI	Right PTA PI	Right PTA RI	Left PTA PI	Left PTA RI
HbA1c	Pearson correlation	-0.001	0.839^*^	-0.092	0.801^*^	-0.071	0.801^*^	-0.102	0.801^*^
	Sig (2-tailed)	0.992	0.000	0.361	0.000	0.484	0.000	0.315	0.000
	N	100	100	100	100	100	100	100	100

## Discussion

T2DM is a metabolic disorder known to increase the risk of diabetic cardiomyopathy and atherosclerotic cardiovascular disease (CVD), which can lead to heart failure through various mechanisms, such as myocardial infarction and chronic pressure overload conditions [8].

According to the recommendations of the American Diabetes Association in 2020, optimal diabetes control is defined by HbA1c levels of <7.0% [9]. A significant indicator of long-term glycemic control, HbA1c reflects the cumulative glycemic history over the preceding two to three months. In this study, HbA1c levels in the study group were within the acceptable range in diabetes treatment and showed an average of 6.63±0.45%, demonstrating relatively well-controlled T2DM.

The current study showed that the mean HbA1c levels were positively correlated with flow resistance of foot arteries (DPA and PTA). This indicated that an increase in HbA1c was associated with increased blood flow resistance. Previous studies demonstrated a linear relationship between the percentage of glycated hemoglobin and micro- and macroangiopathy type complications [10,11]. This might be attributed to chronic hyperglycemia, a predisposing risk factor to diabetes-related angiopathies [12]. The increase in flow resistance might also suggest changes in blood vessel walls (wall stiffening), which affects Doppler flow parameters [13].

On pulsed Doppler, normal waveform of the lower limb arteries was characterized by a triphasic flow pattern. In this study, the characteristic waveforms in the control group are triphasic in 100% of cases, but in diabetes group, the characteristic waveforms are triphasic in 86% of cases and biphasic waveforms in 14% of cases. Consistently, Leoniuk et al. identified triphasic waveforms in 69.6% and biphasic waveforms in 27.7% of well-controlled diabetic patients, while monophasic waveform features were seen in 4% of diabetic individuals [14]. In patients with severe diabetes and intermittent claudication, Shahhen and Sohail reported triphasic flow in as few as 38% of arteries and monophasic flow in as many as 21% of vessels [15].

The present study found that the B-mode diameter and wall thickness of DPA and PTA in type 2 DM were statistically significantly larger when compared to the control group. On the other hand, insignificant differences in lumen diameters were noted in Doppler measurements in both groups. This finding is consistent with Leoniuk et al., who found that the grayscale thickness and diameters of arteries were significantly increased in diabetes group compared to the control group while no significant differences in lumen diameter in color Doppler measurements [14]. Elias et al. reported a strong correlation between type 2 DM and arterial stiffness [16]. The increase in wall thickness and diameters is attributed to vessel wall remodeling due to the development of atherosclerosis. As a result, outward vessel wall remodeling is a compensatory mechanism that allows for the maintenance of a non-narrowed flow lumen while also serving as an early indicator of atherosclerotic lesions [17]. Outward remodeling keeps the lumen normal size in early atherosclerosis. In severe atherosclerosis, blood arteries shrink rather than expand, resulting in inward remodeling [18].

Doppler ultrasound investigations provide accurate blood flow parameters such as RI and PI. The study found that RI and PI have no significant difference between cases and control despite that there was significant increase in vessel wall thickness in diabetes group compared to the control group.

There are certain limitations to our study. First, we made a concerted effort to enroll individuals with well-controlled diabetes; however, there may be selection bias among those who are not. The study is a single-center study, and the sample size is insufficient compared to a multi-center study. This study is still helpful since it gives important data on morphological alterations and Doppler parameters of foot arteries in a preclinical state, which may aid in diabetic patient diagnosis and therapy planning.

## Conclusions

The diabetes group has a considerably larger total diameter and wall thickness of arteries than the control group, indicating thickening of the artery wall as an early indicator of diabetes-related alterations. The RI has a positive linear correlation with HbA1c levels in diabetic patients. The Doppler flow parameters (RI and PI) increased in controlled diabetic without significant difference compared to asymptomatic non-diabetic control group.
